# PDMS/TiO_2_ and PDMS/SiO_2_ Nanocomposites: Mechanical Properties’ Evaluation for Improved Insulating Coatings

**DOI:** 10.3390/nano13101699

**Published:** 2023-05-22

**Authors:** Aldo Cordoba, Eric Mauricio Rivera-Muñoz, Rodrigo Velázquez-Castillo, Karen Esquivel

**Affiliations:** 1 Graduate and Research Division, Engineering Faculty, Universidad Autónoma de Querétaro, Cerro de las Campanas, Querétaro 76010, Mexico; acordoba07@alumnos.uaq.mx (A.C.); rodrigo.velazquez@uaq.mx (R.V.-C.); 2 Center for Applied Physics and Advanced Technology, National Autonomous University of Mexico, A.P. 1-1010, Querétaro 76000, Mexico; emrivera@fata.unam.mx

**Keywords:** insulate materials, mechanical properties, nanocomposites, polymer coatings, reinforcers

## Abstract

The use of nanoparticles (NPs) as reinforcements in polymeric coatings allows for direct interaction with the polymeric chains of the matrix, resulting in a synergistic process through physical (electrostatic forces) and chemical interactions (bond formation) for the improvement of the mechanical properties with relatively low weight concentrations of the NPs. In this investigation, different nanocomposite polymers were synthesized from the crosslinking reaction of the hydroxy-terminated polydimethylsiloxane elastomer. Different concentrations (0, 2, 4, 8, and 10 wt%) of TiO_2_ and SiO_2_ nanoparticles synthesized by the sol-gel method were added as reinforcing structures. The crystalline and morphological properties of the nanoparticles were determined through X-ray diffraction (XRD), Raman spectroscopy, and transmission electron microscopy (TEM). The molecular structure of coatings was through infrared spectroscopy (IR). The crosslinking, efficiency, hydrophobicity, and adhesion degree of the study groups were evaluated with gravimetric crosslinking tests, contact angle, and adhesion tests. It was observed that the crosslinking efficiency and surface adhesion properties of the different nanocomposites obtained were maintained. A slight increase in the contact angle was observed for the nanocomposites with 8 wt% compared to the polymer without reinforcements. The mechanical tests of indentation hardness and tensile strength following the ASTM E-384 and ISO 527 standards, respectively, were performed. As the nanoparticle concentration increased, a maximum increase of 157% in Vickers hardness, 71.4% in elastic modulus, and 80% in tensile strength was observed. However, the maximum elongation remained between 60 and 75%, ensuring that the composites did not become brittle.

## 1. Introduction

Polysiloxanes have been widely used as insulating coatings for power distribution and electrical industries to protect porcelain and glass insulators and conduct cabling from environmental conditions for over five decades [1]. Despite presenting good insulating properties such as a rapid insulating response, high efficiency, low viscosity loss, immunity to depolymerization processes, and rapid application, their performance as coatings is affected by their low mechanical resistance by being exposed to continuous mechanical stress and impacts during handling. This condition triggers mechanical fatigue processes and the formation of leakage currents, electric arcs, and potential discharges (“flashovers”), decreasing their efficiency and lifetime [2].

The addition of reinforcing microstructures as metal oxides has been demonstrated to improve the mechanical response of these coatings. However, the necessity of high concentrations of reinforcers (20–30 wt%) and the low interaction with the polymer have limited the improvement of its mechanical behavior properties [3].

On the contrary, it has been found through mechanical tests and thermal analyses that the mechanical behavior of polymeric coatings can be enhanced with the formation of nanocomposites by incorporating reinforcements with nanometric dimensions [4]. Furthermore, numerous investigations have been carried out, searching to modify other physicochemical properties of polysiloxanes, such as hydrophobicity [5], thermal conduction [4], antibacterial capacity [6], gas permeation [7], antifouling performance [8], and corrosion control [9], with nanocomposites being the main pathway that has successfully allowed the enhancement of these properties.

Adding nanoparticles to the system allows a synergy effect between the nanostructures and the matrix chains by generating a higher contact area, resulting in a more extended interphase zone than conventional reinforcers. These mechanisms influence the formation of new microstructures to improve the stress transfer between the polymer chains or increase the density of mechanical deformation [10,11].

Using metal oxides, specifically titanium dioxide (TiO_2_) and silicon dioxide (SiO_2_), as reinforcers for polymeric matrices has garnered significant interest in recent years. This approach has been explored across a wide range of applications, including but not limited to environmental remediation [12], self-cleaning coatings [13], and protective coatings [14]. Despite the widespread implementation of such composites, most studies focus solely on evaluating the composite behavior without elucidating the underlying mechanisms that drive these observations. Specifically, the changes in the reinforcing matrix interaction, as dictated by variations in chemical species, surface chemistry, particle size, and concentration, remain largely unexplored.

This investigation seeks to not only evaluate the changes in the behavior of composite coatings employed as electrical insulators but also to shed light on the underlying interactions between the polymeric matrix and the TiO_2_ and SiO_2_ reinforcements and to identify the most critical variables (concentration, particle size, and surface properties) that influence the mechanical behavior of the coating. These investigations will be carried out by monitoring the physicochemical effects of using nanoparticle reinforcers on a PDMS matrix.

## 2. Materials and Methods

Before the synthesis, all glassware utilized in the procedure was thoroughly washed with isopropyl alcohol, rinsed with distilled water, and dried with absorbent paper to eliminate potential impurities.

The used reagents for the experimental procedures were isopropyl alcohol (99% Sigma Aldrich, Saint Louis, MO, USA), titanium isopropoxide (TTIP) (97% Sigma Aldrich^®^), ethyl alcohol (Meyer^®^ Mexico City, Mexico), tetraethylorthosilicate (TEOS) (98% Sigma Aldrich^®^), ammonium hydroxide (NH_4_OH) (28% J.T.Baker^®^, Madrid, Spain), dimethyl ketone (99.5%, Meyer^®^) hydroxy-terminated polydimethylsiloxane (PDMS-OH) (2550–3570 cSt, Sigma Aldrich^®^), and dibutyltin dilaurate (DBTL) (95% Sigma Aldrich^®^).

### 2.1. TiO_2_ and SiO_2_ Nanoparticles’ Sol-Gel Synthesis

The synthesis of TiO_2_ nanoparticles was carried out through the sol-gel synthesis method at room temperature, based on the proposed synthesis by [15]. The synthesis began by putting isopropanol (C_3_H_8_O) in a beaker and stirring it under an N_2_ atmosphere for 10 min. Titanium isopropoxide (TTIP) was then slowly added to the solution at a constant rate while stirring, and the mixture was maintained in an inert environment for 20 min. Next, deionized water was added to initiate the hydrolysis reaction, and the resulting solution was stirred for one more hour. The obtained gel was then filtered and dried at room temperature for 24 h. Finally, the dried gel was calcined at 450 °C for 3 h to obtain the anatase crystalline phase [16].

The synthesis of SiO_2_ nanoparticles was performed using the Stöber method [17]. An inert environment (N_2_) was maintained while mixing distilled water and ethyl alcohol (C_2_H_5_OH) for 15 min. Tetraethyl orthosilicate (TEOS) was added dropwise to the solution while stirring for 20 min. A molar ratio of 7:2.7:1 (C_2_H_5_OH:H_2_O:TEOS) was used. The hydrolysis reaction was initiated by adding ammonium hydroxide (NH_4_OH) dropwise until the pH reached 10 and stirring for 20 min. The obtained gel was filtered to eliminate unhydrolyzed residues and dried at room temperature for 24 h (yield reactions are presented in Tables S1 and S2).

The synthesized nanoparticles were subjected to various characterization techniques. Raman spectroscopy was performed using a LabRAM HR spectrometer (HORIBA Jobin Yvon, France) equipped with an NdYHA laser with a wavelength of λ = 523 nm. An X-ray diffraction (XRD) analysis was carried out using a Bruker model D8 Advance X-ray diffractometer (Bruker AXS, Madison, WI, USA) with a CuKα emission anode and a 1.5418 Å wavelength (λ); measurements were performed at 30 kV and 15 mA, with a sweep angle (2θ) from 10° to 80° at a rate of 2° per min. Morphological analyses were conducted using a JEOL JEM 2000FX transmission electron microscope (JEOL USA, Peabody, MA, USA).

### 2.2. Synthesis of PDMS/NPTiO_2_ and PDMS/NPSiO_2_ Nanocomposites

The nanocomposites were prepared using magnetic and ultrasonic stirring techniques following previously published works [18,19]. The materials were obtained through a room-temperature crosslinking reaction between PDMS and TEOS, with dimethyl ketone (C_3_H_6_O) as a solvent and dibutyltin dilaurate (DBTL) as a neutral catalyst. The weight percentages of the reactants were as follows: 58.01% PDMS, 34.87% C_3_H_6_O, 5.80% TEOS, and 1.22% DBTL.

For the incorporation of the NPs as reinforcing agents in the polymer matrix, concentrations of 2, 4, 8, and 10 wt% of TiO_2_ and SiO_2_ were employed to avoid any potential alteration of the dielectric properties of the matrix polymer owing to the presence of the nanoparticles [16]. The nanoparticles were pre-mixed with dimethyl ketone to ensure adequate dispersion with the other constituents of the polymer coating [20].

The chosen concentration of nanoparticles was added to the solvent and stirred at 500 revolutions per minute (rpm) for 20 min. PDMS was added to the solution, and ultrasonic agitation was performed for 25 min using a Bransonic Ultrasonic cleaner model 1510R-DTH (Branson Ultrasonics Corporation, Brookfield, CT, USA). Magnetic stirring was applied for 60 min, followed by adding TEOS while repeating the stirring processes. The DBTL catalyst was added to the solution and stirred at 900 rpm for 120 min.

### 2.3. Nanocomposite Polymeric Characterization Test

Once the composites were synthesized, they were applied to different substrates according to the carried tests. They were applied to metallic (hardness tests), ceramics (adhesion tests), or plastic (contact angle) substrates. Additionally, they were applied in acrylic molds (mechanical stress tests) as test tubes [18]. The solution was placed on the substrate on an utterly horizontal base. The surface was covered to prevent dust from falling, letting it dry at room temperature for 24 h to complete the crosslinking process and solvent evaporation [21].

Crosslinking degree tests were performed with a gravimetric method to determine the weight loss of the materials due to their not-crosslinked chains. Ethanol was used as a solvent for the PDMS composites. Composites were weighed before initiating the test ($P_{i}$). Subsequently, they were submerged in the solvent until they were completely covered for 24 h. The superficial solvent was removed from the composites with absorbent paper, and their weight ($P_{h}$) was recorded. The materials were placed at 60 °C for 2 h to remove the remaining solvent, and the final weight of the coatings ($P_{s}$) was recorded [22].

With this information, the percentage of solvent absorption (A), the weight loss of the coating ($L_{w}$), and the crosslinking degree ($X_{y}$) were calculated through Equations (1)–(3):

(1) $$A=\frac{P_{h}-P_{i}}{P_{s}}\;$$

(2) $$L_{w}=\frac{P_{i}-P_{s}}{P_{i}}\;$$

(3) $$X_{y}=\frac{P_{s}}{P_{i}}\;$$

The contact angle technique of drop-in-air was used to determine the surface hydrophobicity of the composites, taking light contrast photographs and using ImageJ^®^ software (version 1.53e) to determine the contact angle formed by water droplets on the coating surface [23]. Both sides of the surface drop were analyzed to determine the contact angle (θ), taking the average value of the formed angle between the surface and the water drop with ten repetitions for each study group.

The determination of adhesion of the obtained coatings was carried out according to ASTM D3359 by the crosscut method. Three repetitions were performed for each study group, and the results were categorized according to the norm (5A-0A) [24].

The methodology proposed in ASTM E384 was used to determine the indentation hardness of the composites. Vickers hardness was used, providing hardness values in terms of the Vickers Hardness Number (VH) [25]. This test was performed with a Vickers Future-Tech model FM-110 durometer (Future-Tech Corp., Kawasaki, Japan) a 1 kg_f_ load cell, a square pyramidal indenter with an indentation time of 15 s, and an optical microscope with 10× and 40× magnification objectives.

Tensile tests were carried out according to ISO 527-1 using type 5A “dog-bone” test tubes, recommended for crosslinked elastomers. The tests were carried out with a universal testing machine, a 500 N load cell, and a 30 mm/min separation rate. A universal testing machine was used to evaluate the mechanical behavior of the composites and determine their elastic modulus, fracture stress, and maximum elongation [26].

## 3. Results

### 3.1. NPs Characterization

**Figure 1:**
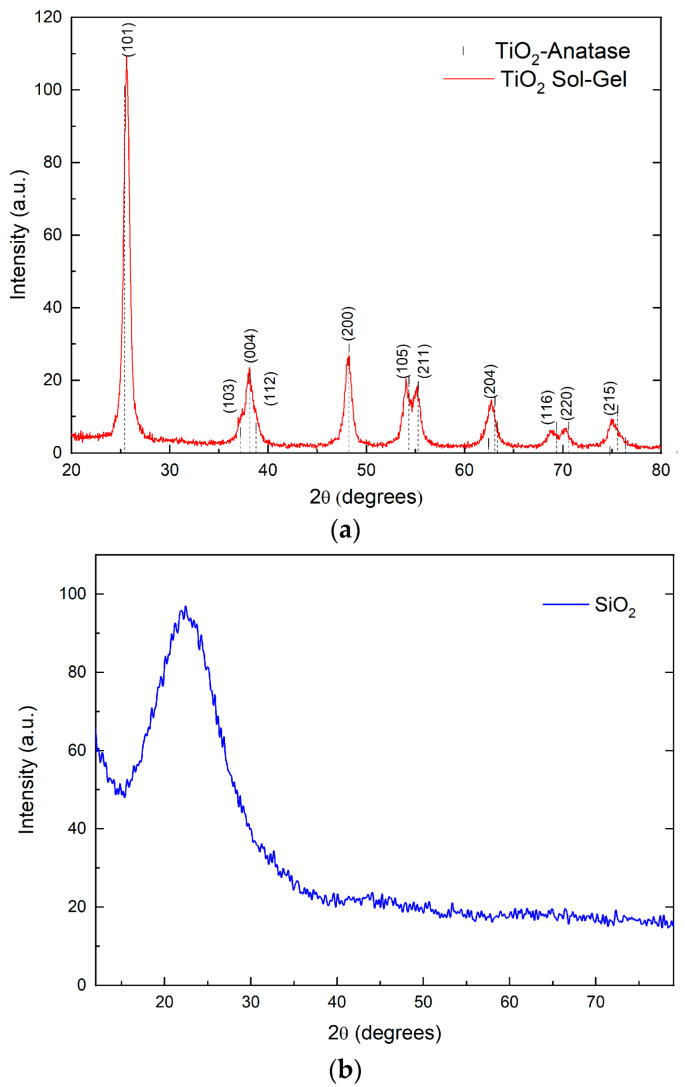
NPs diffractogram spectra of (**a**) TiO_2_ and (**b**) SiO_2_.

Figure 1a shows the diffractogram of the TiO_2_ nanoparticles, which is compared with the JCDPS 01-070-6826 PDF database, corresponding to the anatase crystalline phase of TiO_2_ [16]. The titanium dioxide was obtained, and the diffractogram presents the characteristic Bragg reflections and relative intensities of the anatase according to the PDF mentioned above without displaying any Bragg reflections related to rutile or brookite phases. Figure 1b shows the diffractogram of the synthesized SiO_2_ nanoparticles, observing a material with an amorphous structure. It presents a broad Bragg reflection ranging from 15° up to 30° in 2θ. This diffraction is characteristic of silicon-based materials, presenting a principal contribution from the SiO_2_ quartz-α crystalline phase [27].

Through the X-ray diffractogram of the TiO_2_ nanoparticles, the average crystallite size was estimated using the Williamson–Hall method [28]. The resulting equation from this analysis was y = 0.00683 + 0.01167x with a determination coefficient (R^2^) of 0.765 and an average crystallite size (L) of 22.55 nm. Nanoparticles possess a good enough crystallinity according to the diffractogram.

**Figure 2:**
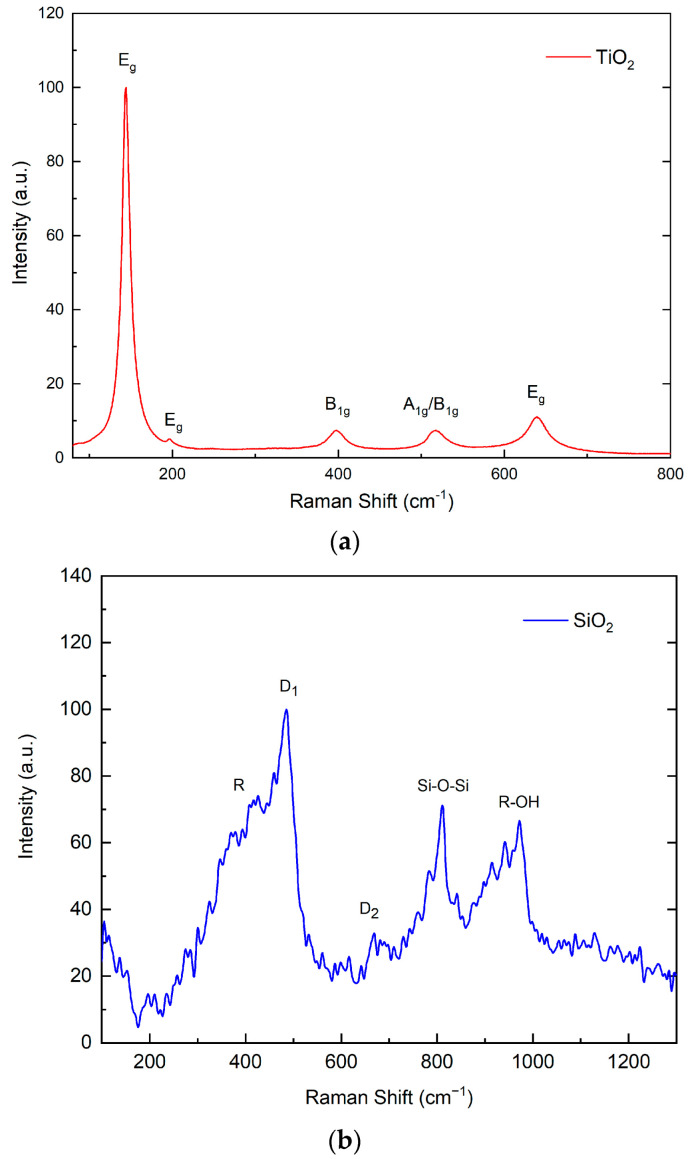
Raman spectra of (**a**) TiO_2_ and (**b**) SiO_2_ nanoparticles.

Figure 2a presents the result from the Raman analysis performed on TiO_2_ nanoparticles. Identifying the six characteristic Raman-active vibrational modes of the anatase tetragonal crystalline phase was possible, corresponding to a D_4h_^19^ space group [29]. These active modes corresponded to the vibrational movement of the Ti and O atoms within the unit cell: A_1g_ (513 cm^−1^) belonged to a vibrational movement of the oxygen atoms, which energetically overlapped with the B_1g_ mode (5134 cm^−1^); the vibrational mode B_1g_ represented the vibrations of Ti atoms. In contrast, the remaining vibrations (E_g_) corresponded to the combined vibrations of O atoms and Ti atoms [29].

Figure 2b presents the Raman spectrum from the SiO_2_ NPs; despite having an amorphous structure, it was possible to identify the characteristic vibrational modes of the system. Five characteristic active vibrational modes of silica were identified: R (410 cm^−1^) represented the bending mode of oxygen atoms in n-atom rings, D_1_ (490 cm^−1^) corresponded to the “breathing” relaxation mode of the 4-atom rings, and D_2_ (605 cm^−1^) was the “breathing” relaxation mode of the 3-atom rings. The vibrational mode within 800 cm^−1^ represented the optical vibration of the O-Si-O system, and the signal at 980 cm^−1^ corresponded to the vibration of the OH- molecule with the silicon atom in the lattice [30].

**Figure 3:**
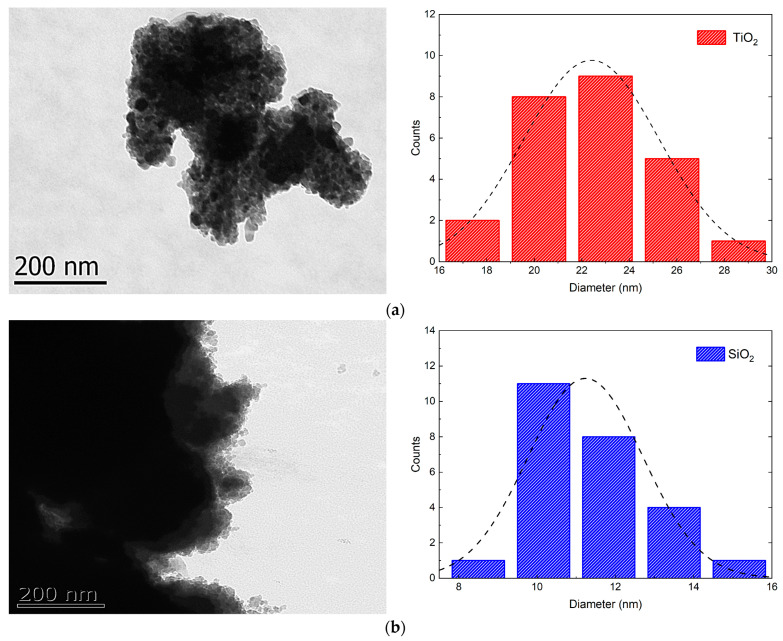
TEM images and particle size distribution of (**a**) TiO_2_ and (**b**) SiO_2_ nanoparticles obtained by sol-gel synthesis.

In Figure 3a, a TEM image of TiO_2_ is presented. The spherical shape and average particle size of TiO_2_ were determined to be 23.7 ± 2.6 nm, confirming the nanoscale size of the system. In Figure 3b, the TEM image of SiO_2_ is also observed to have a spherical morphology. The particle size of SiO_2_ was calculated to be 11.2 ± 1.5 nm, thus confirming that a nanoscale system was obtained too.

**Figure 4:**
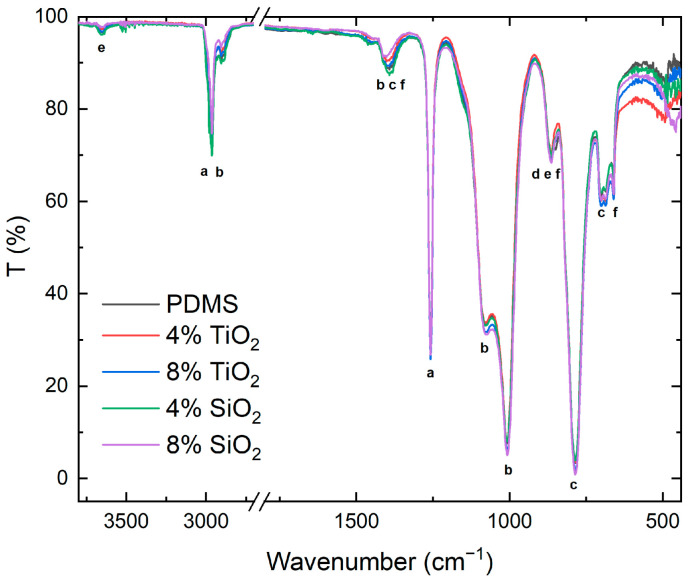
The infrared spectrum of different composites compared to the PDMS material without reinforcers.

Infrared spectroscopy analyses enabled the identification of the reinforcers in the polymeric network and their possible physical and chemical interactions with the polymer matrix. Figure 4 shows the infrared spectra identifying the active vibrational modes of the PDMS crosslinked polymer. Table 1 details the nature of the vibration modes and the chemical bonds to which they correspond [31].

This analysis identified the polysiloxane vibrational modes in each composite sample, observing slight energy modifications of PDMS vibrational modes: 483 cm^−1^, 658 cm^−1^, and 1391 cm^−1^ vibration modes shifted to higher wavenumbers, up to 492 cm^−1^, 661 cm^−1^, and 1402 cm^−1^. This indicates an energy reduction in the vibrational mode, potentially due to a chemical interaction between the polymeric matrix that modified the chemical environment of the composite [32].

At the lower spectrum limit (800 to 400 cm^−1^), it was possible to find the most intense vibrational modes corresponding to TiO_2_ and SiO_2_ nanoparticles: in the range close to 500–450 cm^−1^, a change in the PDMS-10%SiO_2_ composite behavior was observed at 457 cm^−1^, which was related to the vibrational bending mode of the Si–O–Si bond of the SiO_2_ nanoparticles [30]. In the range between 800 and 400 cm^−1^, it was possible to detect a slight increase in the transmittance intensities of the TiO_2_ composites; this area corresponded to a broad vibrational mode corresponding to the Ti–O–Ti bond, characteristic of the anatase TiO_2_ crystalline phase [16].

### 3.2. Composites Evaluation

**Figure 5:**
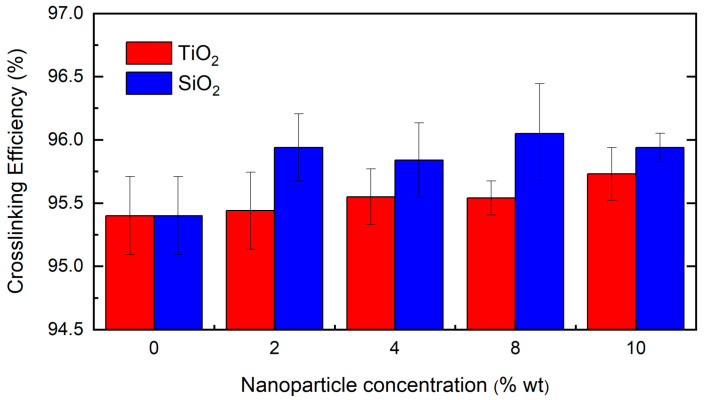
Composite crosslinking performance test.

The crosslinking efficiency of the different polymeric coatings was evaluated to determine if reinforcing nanostructures in the polymeric matrix modifies the degree of crosslinking achieved by the composite. All crosslinked samples weighed around 1.5 g. The initial weight of the films ($P_{i}$), the wet weight ($P_{h}$), and the dry weight after drying in an oven ($P_{s}$) were registered. From this data and Equations (1)–(3), the percentage of solvent absorption (A), the percentage of weight loss of the coating with the solvent ($L_{w}$), and the crosslinking efficiency ($X_{y}$) were calculated. The results of the crosslinking efficiency are presented in Figure 5.

For the solvent absorption (A) evaluation, the results fluctuated from 2.23% to 3.70% for composites reinforced with TiO_2_ nanoparticles. This presumes a proportional trend with the increase in reinforcer concentration, reaching its maximum absorption for the composite with 8% TiO_2_. For the SiO_2_ composites, A ranged between 1.89% and 3.15%, presenting an opposite behavior compared to the TiO_2_, with a minimum value of 10 wt%. The crosslinking efficiency ($X_{y}$) of the different composites did not exhibit a statistically significant variation to that achieved by the PDMS polymer without reinforcers (95.92%) (Table S3). This behavior makes it possible to ensure that the crosslinking efficiency between the polymer matrix and the crosslinking agent is not modified by the presence of the nanostructures in the medium; hence, the hydrophobic, surface, or mechanical properties of these materials will not be modified by this property [33].

**Figure 6:**
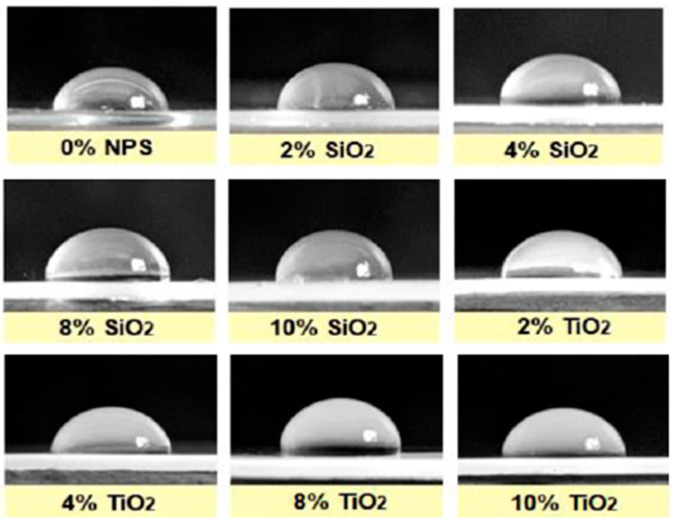
Hydrophobicity tests of PDMS-%NPs nanocomposites through contact angle measurement.

The hydrophobicity was evaluated by measuring the contact angle between a 0.5 mL drop of distilled water and the flat surface of the composites in air, displaying representative images for every composite in Figure 6.

**Figure 7:**
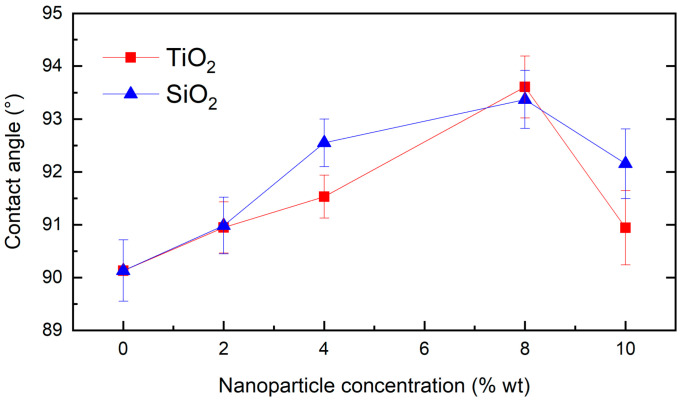
Contact angle measurement for PDMS-TiO_2_ and PDMS-SiO_2_ nanocomposites.

Figure 7 indicates the contact angle measurements from the composites and their standard deviation, clearly observing the behavior and trend for each system depending on the reinforcing nanoparticles (TiO_2_ and SiO_2_) and their concentration (0, 2, 4, 8, and 10 wt%), as well as the comparison to the PDMS polymeric matrix. A maximum contact angle value was presented for both nanocomposites with 8 wt% of the reinforcer. Compared to the pristine PDMS, these composites presented a contact angle of 93.61° and 93.37°, corresponding to an increase of 3.85% and 3.60% for TiO_2_ and SiO_2_, respectively.

Composites with 10 wt% of the reinforcer presented a different behavior. This may happen due to an agglomeration process of the nanostructures in the polymeric matrix, as demonstrated in other research [19]. Agglomeration phenomena of nanoparticles generate a progressive decrease in their surface area, potentially reducing the surface roughness of the composites due to less contribution of nano and microroughness on the surface.

The estimated adhesion by the different coatings on ceramic substrates was evaluated, following the ASTM D3359-09 standard by the crosscut tape test method. Transparent adhesive tape was placed over the covers, parallel to the minor angles formed by the cross. The cut marks left on this tape after the adhesion tests were studied. The results are recorded in Table 2.

All of the study groups exhibited similar behavior. Detachment was observed only where the incisions were made, corresponding to a 4A classification. The property of the composites did not present a perceptible variation that denoted a modification in the adhesion performance due to the presence of the nanoparticles in the polymeric matrix [24].

The indentation hardness tests were conducted based on the ASTM E-384 standard. The measurements were made on the coating with dimensions of 20 × 20 × 0.4 mm supported on a metallic substrate with three replicates for each study group. Table 3 shows the calculated values and standard deviation of indentation hardness of the nanocomposites. The progressive change of the indentation hardness was observed depending on the concentration and kind of nanoparticle.

All composites exhibited a gradual and directly proportional increase in indentation hardness as the reinforcer concentration increased. The best results corresponded to the composites with SiO_2_ reinforcers, among which 10 wt% stood out. It presented a Vickers hardness of 11.45 ± 0.45 kgf/mm^2^. It represents an increase of 157% compared to the hardness of PDMS without reinforcers (4.45 ± 0.104 kgf/mm^2^).

**Figure 8:**
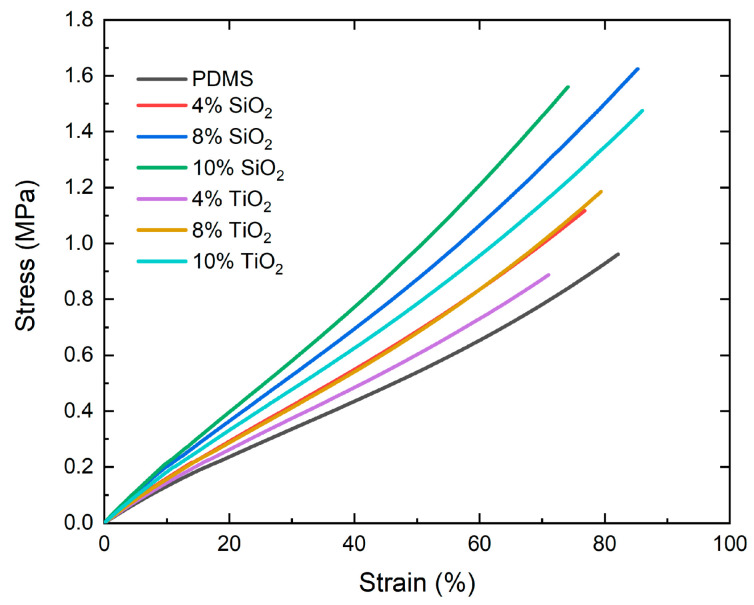
Tensile strain tests of PDMS nanocomposites.

The mechanical tensile tests were performed based on ISO 527-1. The proper tests were conducted for the six study groups with the highest reinforcer concentration (4, 8, and 10 wt%) and the PDMS coating without NPs (0 wt%). Each group was measured with five repetitions. Figure 8 shows the average stress–strain tests of the different composites. A progressive increase was observed in the deformation resistance of the composites concerning the reinforcer concentration up to 8w%t for SiO_2_ and 10 wt% for TiO_2_. Despite this resistance increment, the mechanical behavior of the composites maintained the characteristic PDMS elastomeric behavior [34].

The applied tensile stress test results are presented in Table 4, where the average and dispersion of the elastic modulus values, the maximum elongation percentage, and the fracture stress of the different materials are registered.

The elastic modulus enhanced progressively as the concentration of nanoparticles increased, reaching a maximum value of 2120 ± 0.036 MPa for nanocomposites with TiO_2_ and 2453 ± 0.104 MPa for SiO_2_ with a 10 wt% concentration. This modification represented an increase of 48.25% and 71.4%, respectively, compared to the PDMS without any reinforcer (1.43 MPa).

The maximum elongation obtained for the composites differed from that presented by the elastic modulus. Despite the different nanoparticles and concentrations, the maximum elongation of the composites was within the standard deviation of PDMS without reinforcers. These variations were not statistically significant, maintaining values of maximum elongation between 70% and 85%.

In fracture stress, the results presented similar behavior to that found in the elastic modulus, with an increase in the maximum fracture stress proportional to the increase in reinforcer concentration. In the SiO_2_ nanocomposite, a tendency to decrease the maximum stress was reached when evaluating the highest concentration (10 wt%) without being statistically significant. The 8 wt% SiO_2_ composite presented the highest average tensile strength (1.623 ± 0.145 MPa), corresponding to an 80% increase compared to PDMS without reinforcers.

## 4. Discussion

The structural properties of the material were evaluated by analyzing the TiO_2_ diffractogram. The results indicated that the applied thermal process led to forming a single-phase material with an anatase crystal structure, consistent with previous findings [16]. A further analysis revealed that the sol-gel synthesis technique resulted in a nanostructured system with minimal mechanical stresses in its unit cell. This result was supported by the observation that the Bragg reflections showed no shift and exhibited a symmetrical normal distribution for the maximum diffraction intensity. Micro-tensions’ low contribution to the broadening of the diffraction signals was inferred from the full width at half maximum (FWHM) of the plane reflections and the calculation of the crystallite size. This indicates that the synthesis process is primarily responsible for the observed low mechanical stresses. Specifically, the absence of temperature or pressure precludes the generation of mechanical stress or deformation [35].

The presence of characteristic vibrational modes of the TiO_2_ anatase crystalline phase, combined with XRD analyses, confirms the synthesis of a single-phase crystalline nanomaterial. Furthermore, the expected energy distribution in these vibrational modes confirms a network with minimal cell stresses and deformations. In the case of SiO_2_ particles, despite having an amorphous structure as determined by X-ray diffraction, Raman spectroscopy allows the analysis of the present chemical species and identification of the material. Although with more significant variation than a crystalline system, the presence of different chemical species, such as Si-O and Si-OH, characteristic of a silica system, can be determined. Furthermore, the presence of the D_2_ vibrational mode indicates the non-porous structure of the nanostructured silica. This observation agrees with the methodology used, which involved an alkaline catalyst [30].

TEM analyses were conducted to verify the synthesis of TiO_2_ and SiO_2_ nanoparticles. Notably, there were significant differences between both particles, primarily their size. SiO_2_ particles have a size that is less than half of that of TiO_2_ particles, resulting in a potentially increased surface area and a greater chance for polymer–reinforcement interaction compared to titanium. Furthermore, agglomeration seems to occur strongly in SiO_2_ particles, potentially due to a higher surface energy because of its smaller size [36].

Through an IR analysis, the modifications of the PDMS vibrational modes were identified. The wavenumber reduction of some of the main polymer matrix chain bond vibrations (CH_3_, and -Si(CH_3_)_2_-O-Si(CH_3_)_2_) corresponds to a reduction in their oscillation energy, potentially due to a chemical interaction with the nanoparticle reinforcers, which modifies the chemical environment of the composite [32].

The non-alteration of the crosslinking efficiency makes it possible to ensure that the crosslinking efficiency between the polymer matrix and the crosslinking agent is not modified by the presence of the nanostructures in the medium; hence, the hydrophobic, surface, or mechanical properties of these materials will not be modified by this property [33].

The contact angle enhancement with the presence of the reinforcers might be due to an increase in the surface area of the coatings caused by a superficial roughness rise, which depended on the generation of a larger surface area in the material as the concentration of particles increased [5]. In addition, for the composites of higher concentrations (10 wt%), agglomeration processes of the nanoparticles may occur, triggering a progressive decrease in the surface area of the nanostructures and, consequently, a roughness reduction in the coating [19].

The indentation hardness enhancement, corroborated with the IR analysis, has been proposed due to the interaction between reinforcers and the polymeric matrix. The interaction, whether chemical (bond formation) and/or physical (Van der Waals and hydrogen bonds), modifies the sliding processes of the polymer chains, reducing their mobility during mechanical stress or thermal processes [4]. Likewise, obtaining a similar progressive enhancement in the elastic modulus is related to the indentation hardness behavior. Plastic deformation in the material requires sufficient stress to overcome this elastic region of the composites during the assays [33].

The “stagnation” process observed in fracture stress has been analyzed in different micro and nanometric reinforcements. As the concentration of a reinforcer in a polymeric matrix increases, the mechanical resistance may even begin to decrease. By raising the concentration of nanoparticles in the polymer matrix, the probability of interaction between them increases due to their high surface energy, causing them to agglomerate. The agglomeration generates a particle size increase in the reinforcers; hence, a reduction in the available interaction area and, therefore, a reduction in the reinforcement effect is induced [3].

Despite using the sol-gel synthesis method for both reinforcers, the thermal process on the TiO_2_ nanoparticles can be a differential factor. This process generates a chemical modification on the nanoparticle surface: the hydroxyl bonds (-OH) resulting from the gel formation process are removed during calcination [16], unlike SiO_2_, which maintains these functional groups (silanol) on the surface [30]. This surface modification seems to reduce the interaction of the TiO_2_ reinforcers compared to the SiO_2_ nanoparticles, observing a better mechanical performance for this study group.

## 5. Conclusions

In conclusion, the successful synthesis of nano-reinforced coatings (SiO_2_ and TiO_2_) has been achieved with improved mechanical properties, including indentation hardness and tensile strength, depending on the concentration and type of reinforcer. The maintenance of crosslinking, hydrophobic, and surface adhesion properties, which are essential for insulating coatings, has also been demonstrated.

The interaction between the polymer and nanoparticles has been shown to have a significant impact on the mechanical properties, with coatings containing 8 wt% SiO_2_ and 10 wt% TiO_2_ exhibiting the best mechanical resistance. Notably, the PDMS-8%SiO_2_ composite has demonstrated the best mechanical response, with up to 157% improvement in indentation hardness (Vickers hardness) and an 80% increase in fracture tensile stress.

Improving the mechanical response of PDMS composite polymer coatings while maintaining the same ductile behavior of the polymer (elastomer) is essential for their application in the electrical insulator industry. This potential improvement in the lifespan of such coatings without compromising the fragility of the crosslinked material highlights the importance of nanostructures in reinforcing polymeric materials for future applications. 

The results showed a significant improvement in the mechanical behavior of the composites; certain limitations were identified, including the agglomeration of nanoparticulate reinforcers and limited chemical interaction with the polymeric matrix. The surface chemistry, particle size, and dispersion of the reinforcement properties appear to be critical factors affecting the interaction with the polymer matrix. Further studies are necessary to overcome these limitations and optimize the mechanical properties of the composites.

## Figures and Tables

**Table 1:** Active vibrational modes observed in infrared spectroscopy of PDMS [31].

	Vibrational Bond	Wavelength (cm^−1^) *
a	-SI(CH_3_)_n_	2905–2960
		1280–1255
b	-Si(CH_3_)_2_-O-Si(CH_3_)_2_-	2905–2960
		1390–1410
		1100–1000
c	Si-(CH_3_)_3_	1410
		850–730
		730–650
d	Si-(CH_3_)_3_	850–840
		765
		715–680
e	Si-OH	3640–3695
		810–960
f	Si-CH_3_	1410
		750–870
		730–650

* The displayed wavenumber values indicate the energy range in which these active vibration modes appear. The energy fluctuates depending on other system properties, such as molecular weight.

**Table 2:** Adhesion test results for PDMS nanocomposites.

Composites	Observations
PDMS	* 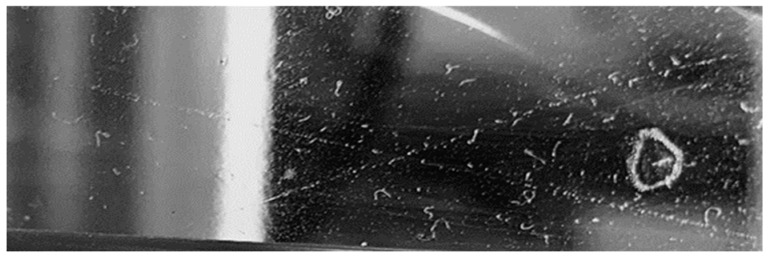 *
2% TiO_2_	* 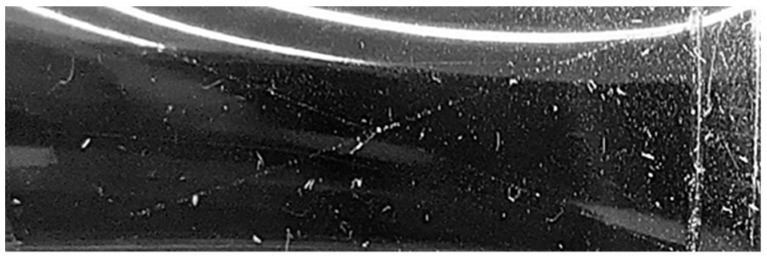 *
2% SiO_2_	* 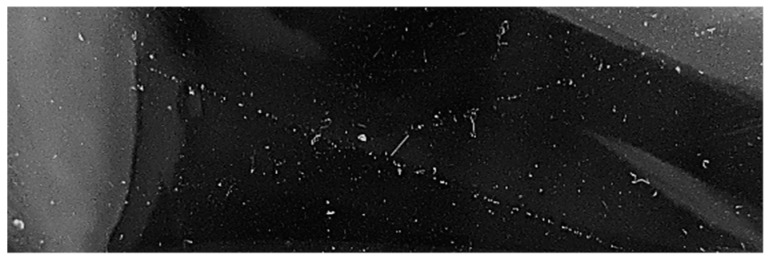 *
4% TiO_2_	* 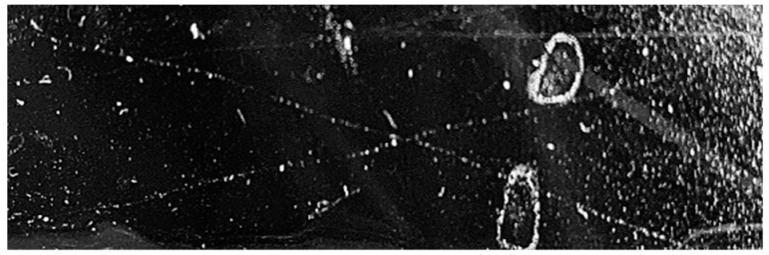 *
4% SiO_2_	* 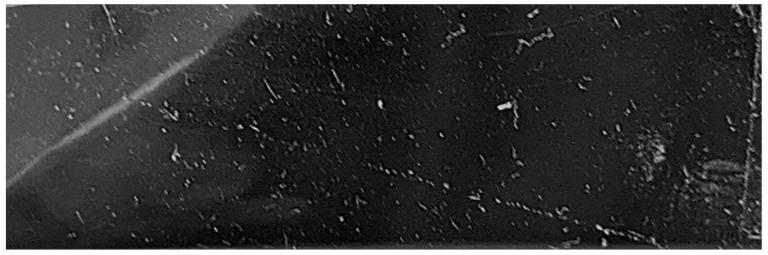 *
8% TiO_2_	* 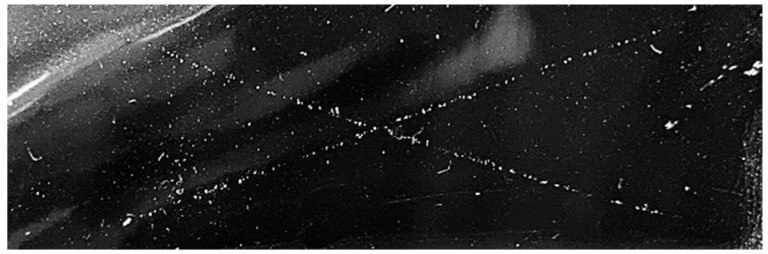 *
8% SiO_2_	* 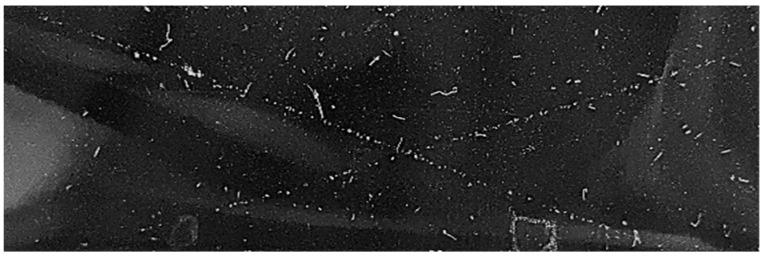 *
10% TiO_2_	* 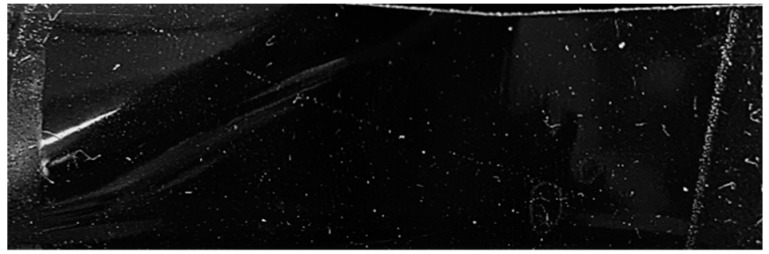 *
10% SiO_2_	* 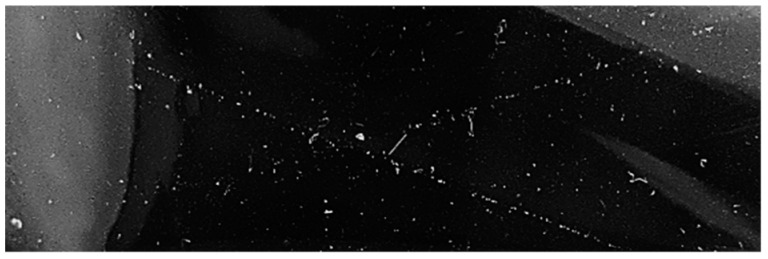 *

**Table 3:** Vickers hardness tests’ results for the different nanocomposites.

Reinforcement	Wt%	Vickers Hardness (kg/mm^2^)
PDMS	----	4.45 ± 0.104
TiO_2_	2%	5.70 ± 0.201
	4%	6.14 ± 0.054
	8%	6.58 ± 0.338
	10%	6.93 ± 0.245
SiO_2_	2%	6.63 ± 0.215
	4%	7.04 ± 0.239
	8%	8.34 ± 0.172
	10%	11.45 ± 0.450

**Table 4:** Mechanical properties of PDMS nanocomposites from tensile stress tests.

Group	Elastic Module (MPa)	Fracture Stress (MPa)	Maximum Elongation (%)
PDMS	1.430 ± 0.135	0.900 ± 0.087	79.240 ± 6.920
4% TiO_2_	1.797 ± 0.064	0.890 ± 0.010	66.667 ± 1.477
8% TiO_2_	1.883 ± 0.067	1.083 ± 0.094	73.117 ± 5.531
10% TiO_2_	2.120 ± 0.036	1.323 ± 0.138	79.467 ± 5.902
4% SiO_2_	1.850 ± 0.087	0.930 ± 0.165	66.230 ± 9.205
8% SiO_2_	2.323 ± 0.023	1.623 ± 0.145	83.537 ± 5.302
10% SiO_2_	2.453 ± 0.104	1.383 ± 0.157	67.407 ± 5.860

## Data Availability

No new data were created or analyzed in this study. Data sharing is not applicable to this article.

## Supplementary Materials

The following supporting information can be downloaded at: https://www.mdpi.com/article/10.3390/nano13101699/s1. Table S1: Reaction efficiency of the sol-gel method for TiO_2_ nanoparticles; Table S2: Reaction efficiency of the sol-gel method for SiO_2_ nanoparticles; Table S3: Composite crosslinking performance results.
