# Supplementary material for: PDMS/TiO2 and PDMS/SiO2 Nanocomposites: Mechanical Properties’ Evaluation for Improved Insulating Coatings
Source: Nanomaterials (Basel). 2023 May 22;13(10):1699. doi: 10.3390/nano13101699 (PMC10223323; doi:10.3390/nano13101699)
Supplement: Supplementary file 1 [file nanomaterials-13-01699-s001.zip › nanomaterials-2397596-supplementary.pdf]

nanomaterials

Supplementary Materials

PDMS/TiO_2 and PDMS/SiO_2 Nanocomposites: Mechanical Properties' Evaluation for Improved Insulating Coatings

Aldo Cordoba [1], Eric Mauricio Rivera-Muñoz [2], Rodrigo Velázquez-Castillo [1] and Karen Esquivel [1,*]

[1] Graduate and Research Division, Engineering Faculty, Universidad Autónoma de Querétaro, Cerro de las Campanas, Querétaro 76010, Mexico; acordoba07@alumnos.uaq.mx (A.C.); rodrigo.velazquez@uaq.mx (R.V.-C.)
[2] Center for Applied Physics and Advanced Technology, National Autonomous University of Mexico, A.P. 1-1010, Querétaro 76000, Mexico; emrivera@fata.unam.mx
* Correspondence: karen.esquivel@uaq.mx; Tel.: +52-442-192-12-00 (ext. 65401)

Table S1. Reaction efficiency of the sol-gel method for TiO_2 nanoparticles.

#	Date	C_3H_7OH (ml)	$C_{12}H_{28}O_4Ti$ (ml)	H_2O (ml)	TiO_2 (g)
1	21/09/2020	142	14	142	3.643
2	12/10/2020	142	14	142	3.489
3	21/06/2021	142	14	142	3.895

Table S2. Reaction efficiency of the sol-gel method for SiO_2 nanoparticles.

#	Date	H_2O (ml)	C_2H_5OH (ml)	TEOS (ml)	SiO_2 (g)
1	22/09/2020	1.2	4 ml	3.78	0.316
2	23/09/2020	2.4	8 ml	7.56	0.661
3	12/10/2020	3.6	12 ml	11.3	2.543
4	23/06/2021	7.1	24 ml	22.6	4.102

Table S3. Composite crosslinking performance results.

NPs (%)	TiO_2 (%)		SiO_2 (%)	
0	95.40	± 0.31	95.4	± 0.31
2	95.44	± 0.30	95.94	± 0.25
4	95.55	± 0.22	95.84	± 0.29
8	95.54	± 0.13	96.05	± 0.39
10	95.73	± 0.21	95.94	± 0.11
